# Erratum: Measurement of transverse emittance and coherence of double-gate field emitter array cathodes

**DOI:** 10.1038/ncomms14526

**Published:** 2017-02-24

**Authors:** Soichiro Tsujino, Prat Das Kanungo, Mahta Monshipouri, Chiwon Lee, R. J. Dwayne Miller

Nature Communications 7: Article number: 13976; DOI: 10.1038/ncomms13976 (2016); Published 12 23 2016; Updated 02 24 2017

An incorrect version of the Supplementary Information was inadvertently published with this Article where wrong vector notation was used for equation (4), wrong font for ‘phi' on page 10 and the wrong unit ‘(m)' was used instead of ‘(mm)' for the *x*-axis of Supplementary Fig. 2a. The HTML has now been updated to include the correct version of the Supplementary Information.

Also, the Transparent Peer Review file was inadvertently published. This has now been removed.

