# Supplementary material for: Measurement of transverse emittance and coherence of double-gate field emitter array cathodes
Source: Nat Commun. 2016 Dec 23;7:13976. doi: 10.1038/ncomms13976 (PMC5196429; doi:10.1038/ncomms13976)
Supplement: Supplementary Information — Supplementary Notes, Supplementary Figures and Supplementary References [file ncomms13976-s1.pdf]

**Supplementary Note 1: Evaluation of the transverse coherence length**

The rms diffraction spot size $D$ (equal to $\Delta^{(1)}/(2\sqrt{2\ln 2})$ in the main text) on the screen is a function of the rms transverse coherence length $\sigma_c$ of the incident beam on the sample, the rms beam spot size $\sigma_b$, the sample domain size $\sigma_s$, and the magnification ratio $M$ (approximately 3 in the condition of Figure 5) of the sample size on the screen. When $\sigma_s$ is much smaller than $\sigma_b$ but larger than $\sigma_c$, $D$ will be determined by $\sigma_s$ and $\sigma_c$. When $D$ is much smaller than $M\sigma_s$, $D$ will be solely determined by $\sigma_c$. When $D$ is comparable to $M\sigma_s$, $D$ gives the lower limit of $\sigma_c$. In the experimental condition of Figure 5, $D$ is more than factor of 2 smaller than $\sigma_b$. Therefore the value of $\sigma_c$ estimated from the comparison of $(R/\Delta^{(1)})a_{gp}$ with the model calculation is the lower limit.

The model calculation used for this comparison is the following. We neglected one of the transverse direction ($y$-direction) without loss of generality, and assumed the incident electron beam on the sample at $z = 0$ plane as the random summation of Gaussian wave packets with the rms transverse momentum of $\sigma_p$, and the corresponding rms transverse coherence length of $\sigma_c$ as,

$$\Psi(z,x) = e^{ikz}\sum_s \psi(x - x_s) \;; \qquad (1)$$

$$\psi(x) \sim \int dp e^{-p^2/(2\sigma_p^2)} e^{ipx/\hbar} = \text{const.} \times e^{-x^2/(2\sigma_c^2)} \;; \qquad (2)$$

where

$$\sigma_c = \frac{\hbar}{\sigma_p} = \frac{\hbar}{mc}\left(\frac{\sigma_p}{mc}\right)^{-1} = \frac{\hbar}{mc}\left(\frac{\varepsilon_x}{\sigma_{s,x}}\right)^{-1} . \qquad (3)$$

In Supplementary Equation (1-2), $\hbar k \gg \sigma_p$ was implicitly assumed. Random distribution of $x_s$ (within the range of $x < \sigma_b$) assures the uniform beam intensity on the sample. In Supplementary Equation (3), $\varepsilon_x$ is the intrinsic emittance (normalized rms, in the $x$-direction) and $\sigma_{s,x}$ is the source size (rms), thus in agreement with equation (6) in the main text, when the spot size on the sample is same as the source size that was fulfilled in the LEED experiment.

The wave function of the electrons by an atom located at $x = na$ by the incident plane wave exp(i$kz$) is written as,

$$\psi(\vec{r}) \sim \alpha(\theta)\frac{e^{ik|\mathbf{r}-\mathbf{r}_n|}}{|\mathbf{r}-\mathbf{r}_n|}; \quad (4)$$

where $\mathbf{r}_n = (na, 0, 0)$ and $\alpha(\theta)$ is the scattering amplitude of the plane wave at the angle $\theta$ away from the beam axis. The number of the atoms near that contributes the diffraction by the wave $\psi(x - x_s)$ with $x_s = 0$ is restricted by $\sigma_c$. On the screen at $z = L$, the diffracted intensity $S(x)$ as a function of $x$ can be written by only considering the phase factor with good approximation as,

$$S(x_s) \sim \left| \sum_{n=-\infty}^{\infty} \alpha(\theta) \exp\left[ -2\pi i \frac{na}{\lambda} \frac{x_s}{\sqrt{L^2 + x_s^2}} - \frac{(na)^2}{2\sigma_c^2} \right] \right|^2. \quad (5)$$

In Supplementary Equation (5), $\lambda = 0.387$ Å is the wavelength of the electron at 1 keV. Accordingly, the sharpness of the diffraction spots is inversely proportional to $\sigma_c$ and the number of the contributing unit cells (see Supplementary Reference 1). To quantitatively compare with experiment, we calculated for the 1$^{st}$ order diffraction spot the ratio of the spot width (full width at the half maximum) to the spot position from the center of the screen as a function of $\sigma_c/a_{gp}$ in the limit of small sample size and/or the incident beam size for the condition that approximates the experimental condition of Figure 5: the beam energy of 1 kV, and the sample-screen distance $L$ of 26.4 mm. The value of $\alpha(\theta)$ was obtained from the elastic scattering cross-section of 1 kV electron by carbon atom from the NIST database [Supplementary Reference 2]. Supplementary Figure 1 shows the calculated result: the relation between $R/\Delta^{(1)}$ and $\sigma_c/a_{gp}$. From the experimentally observed ratio, $R/\Delta^{(1)}$ equal to 13.7±3.5 and Supplementary Figure 1, we evaluated $\sigma_c = (3.6 \pm 1.0)\, a_{gp} = 0.89 \pm 0.25$ nm. As discussed above, this is the lower estimate of the $\sigma_c$ with the present method. As discussed in the main text, this is compatible with the value calculated from the intrinsic emittance.

Because of the finite potential difference between the graphene sample and the entrance of the electron detector, the reflection from the graphene is radially stretched: the deceleration of the longitudinal motion elongates the radial propagation distance. However, since both $R$ and $\Delta^{(1)}$ were stretched, the influence on the ratio ($R/\Delta^{(1)}$) was small. We simulated this effect with the experimental condition of Figure 5 and confirmed that the influence was indeed small, as summarized in Supplementary Figure 2. In free propagation case, the estimated radial

position of the 1st order diffraction spots on the screen was approximately 4 mm, that is 30% smaller than the observation of $R \cong 6$ mm. However, as expected the influence on the evaluation of $\sigma_c$ by this deceleration was less than a few % therefore negligible.

**Supplementary Note 2: Influence of the beam potential and beam collimation on the diffraction**

Supplementary Figure 3 shows LEED results from the second graphene sample with the graphene voltage equal to 600 V and 800 V, respectively for Supplementary Figure 4 (a) and (b). This was also measured with 50 V entrance plane potential of the electron detector. As expected, the distance of the diffraction spots from their apparent center (marked by crosses) is larger for 600 V case than that for 800 V because of the longer wavelength and the larger reflection angle at the lower beam potential. The ratio of the distance of the diffraction spots from their apparent center $R_{600V}/R_{800V}$ is equal to ~1.25 and equal to the inverse of the ratio of the potential, $(800V/600V)^{1/2}$ = 1.16 within 8 %. The deviation partly originates from the deceleration as described above. The 2$^{nd}$ order reflection with the smaller longitudinal was not observed because those were deflected away to the outside of the detector area.

In Supplementary Figure 4, we show the three diffraction images with $k_{col}$ equal to 0.99, 0.91, and 0.86 observed with the same sample and measurement condition as Supplementary Figure 3 (the sample voltage was 800 V and the detector entrance potential was 50 V). The broadening of the diffraction spots with the decrease of $k_{col}$, hence with the increase of the angular divergence, is apparent and consistent with the fact that the transverse velocity spread and energy is larger at smaller $k_{col}$ as demonstrated in the emittance measurement. We found that the diffraction spot size increased by a factor of 2 when $k_{col}$ was decreased from 0.99 to 0.86.

**Supplementary Figure 1:** Calculated relation between the $R/\Delta^{(1)}$ and the ratio of the rms transverse coherence length $\sigma_c$ normalized by $a_{gp}$, where $R$ is the distance of the 1st order diffraction spot from the beam axis, $\Delta^{(1)}$ is the spot width (FWHM) of the diffraction spot, and $a_{gp}$ is the lattice constant of graphene.

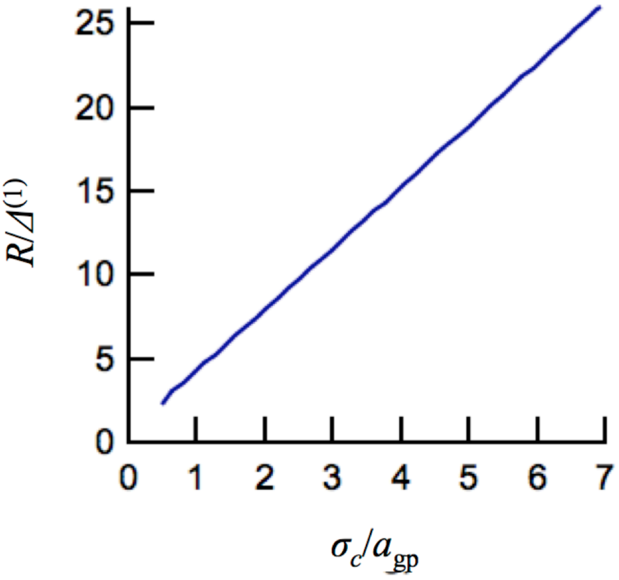

**Supplementary Figure 2**: (a) The orbit of 1 keV electron reflected by the graphene by 9°, that corresponds to the 1st order diffraction by the graphene, propagating by 27 mm to the electron detector with the entrance potential of 250 V. (b) The relation of the radial position on the screen: *xs, free* = free propagation, and *xs, expanded* = deceralated propagation with the detector potential of 250 V. (c) The 1st order diffraction peak under the deceralated propagation condition in Figure 5, calculated when $\sigma_c$ was equal to 3.6 $a_{gp}$.

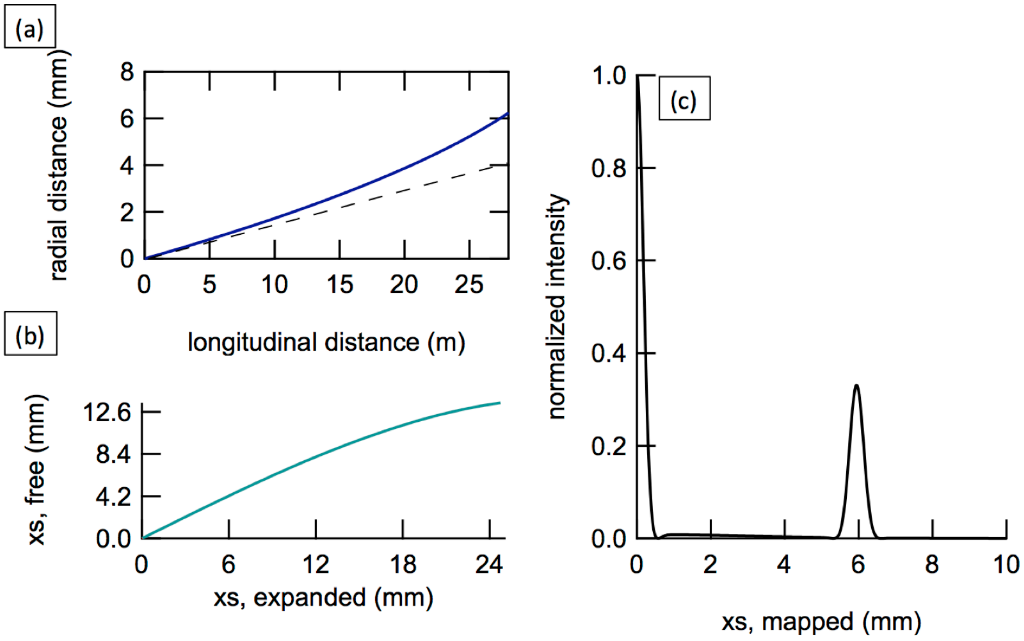

**Supplementary Figure 3:** LEED from suspended graphene by double-gate FEA beam with $k_{col}$ = 0.99, when the beam potential was (a) 500 V, and (b) 800 V. The beam potential was set by applying the voltage to the graphene. A cross denotes the center of the diffraction spots. The lines indicate the distance from the center to the diffraction spots. The scale bar is 6 mm on the screen.

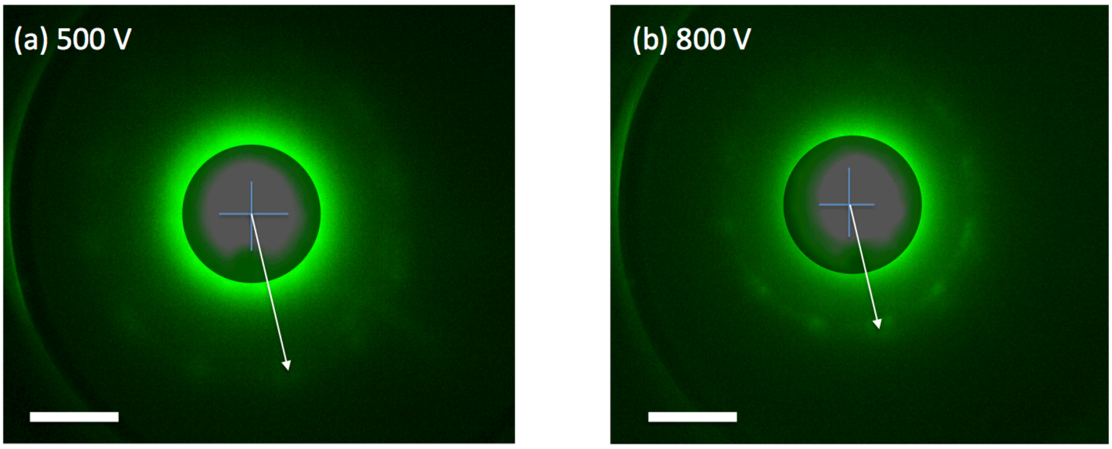


**Supplementary Figure 4**: The influence of $k_{col}$ and beam collimation on the diffraction. (a)-(c) shows the result with $k_{col}$ = 0.99, 0.91, and 0.96, respectively, at $V_{ge}$ = 43 V and the sample potential of 800 V. Other conditions were same as Supplementary Figure 3.

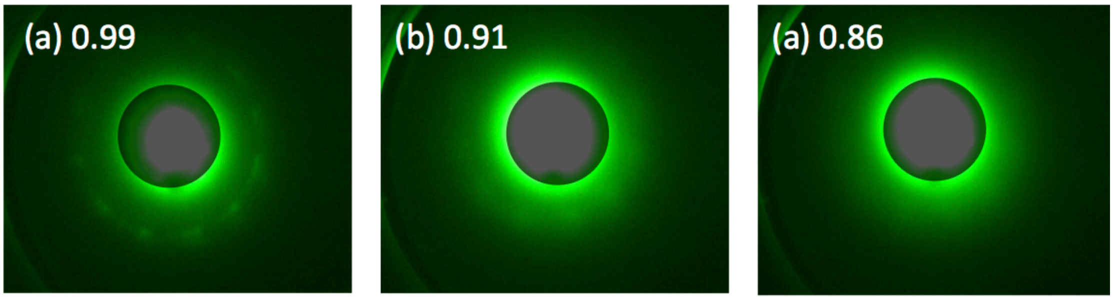

**Supplementary Note 3: Theory of the intrinsic emittance and the average transverse energy of field emission beam**

Here we describe the derivation of the average transverse energy of field emission beam emitted from a free-electron metal. The field emission current density $J$ is calculated for a free electron metal with the Fermi energy $E_F$ an the work function $\phi$ much lager than the temperature $T$ as the integration of the current density per energy $j(E)$ by the total electron energy $E$ [Supplementary Reference 3,4],

$$J = \int dE j(E) \quad . \tag{6}$$

The current density $j(E)$ is in turn given by the Fermi-Dirac distribution $f(E)$ and the transmission function $T(E_z)$ given as a function of the normal energy $E_z$ (the energy in the direction normal to the emission surface),

$$j(E) \sim f(E) \int^{E} dE_z T(E_z) \quad ; \tag{7}$$

$$f(E) = \frac{1}{e^{(E-E_F)/k_B T} + 1} \quad . \tag{8}$$

The transmission function $T(E_z)$ is written as [Supplementary Reference 5],

$$T(E_z) = P(E_z) \exp\left[-G(E_z)\right] \quad ; \tag{9}$$

where the prefactor $P(E_z)$ is a weakly varying function of $E_z$ and neglected in the following discussion as described in the main text. The exponent $G(E_z)$ in Supplementary Equation (9) is calculated by the Jeffreys-Wentzel–Kramers–Brillouin (JWKB) approximation of the Schottky barrier including the image charge. In the absence of the image potential, the phase factor $G_0(E_z)$ at $E_F$ is written as [equation (2.4) in Supplementary Reference 5],

$$G_0(E_F) = b\phi^{3/2} / F_{tip} \quad . \tag{10}$$

In Supplementary Equation (10), $b$ is a constant equal to 6.830890 $eV^{-3/2}$ V $nm^{-1}$ by the work function $\phi$ equal to ~4.5 eV for Mo and the electric field $F_{tip}$ equal to ~(2-5) GV $m^{-1}$ in typical field emission case.

When the image potential is taken into account, the calculation of $G(E_z)$ involves elliptic integrals. Following Forbes and Deane [Supplementary Reference 5] of their recently reported expression $G(E_F)$ is written as,

$$G(E_F) = \upsilon_F G_0(E_F) \ . \tag{11}$$

$$\upsilon_F = 1 - f_F + (f_F / 6)\ln f_F \ . \tag{12}$$

$$f_F = c^2 F_{tip} / \phi^2 \ . \tag{13}$$

where $c$ is a constant equal to 1.199985 eV $V^{-1/2}$ $nm^{1/2}$ (not to be confused with the speed of light). The calculation of the integral in Supplementary Equation (7) is performed with an approximation in which $G(E_z)$ is linearly expanded near $E_F$ ,

$$G(E_z) \approx G(E_F) + (E_z - E_F)/d_F \ ; \tag{14}$$

$$\frac{1}{d_F} = \left.\frac{\partial G}{\partial E_z}\right|_{E_z=E_F} = \tau_F \left.\frac{\partial G_0}{\partial E_z}\right|_{E_z=E_F} = \tau_F \frac{3}{2}\frac{b\phi^{1/2}}{F\mathrm{tip}} \ ; \tag{15}$$

$$\tau_F = \upsilon_F + \frac{3\phi}{2}\frac{\partial \upsilon_F}{\partial \phi} \ . \tag{16}$$

The factor $\tau_F$ in Supplementary Equations (15-16) is close to 1. By substituting Supplementary Equations (9-16) to (7), the Fowler-Nordheim equation in Forbes-Deane form is obtained,

$$J \sim F_{tip}^{2-\delta} \exp\left[-b\phi^{3/2} / F_{tip}\right] \ . \tag{17}$$

In Supplementary Equation (17), the correction factor $\delta$ (approximately equal to 0.8) of the exponent of the $F_{tip}$ in front of the exponential results from the high precision expansion of $\upsilon_F$. In conventional, less accurate approximations, $\upsilon_F$ is approximated by $0.95 - f_F$ that gives $\delta$ equal to 0.

In the case of a metal with the work function $\phi$ equal to 4.5 eV and the $F_{tip}$ equal to 2-5 GV/m, $d_F$ is in the order of 0.1-0.2 eV and much smaller than $E_F$. Therefore the linear approximation of Supplementary Equation (14) is justified.

Now by using the integrand of Supplementary Equation (6) as the distribution function, the average of $E$ and $E_z$ can be calculated. Following Swanson et al. in Supplementary Reference 6, first we define the deviation of $E$ and $E_z$ from $E_F$,

$$\varepsilon = E - E_{\mathrm{F}} \ ; \tag{18}$$

$$\varepsilon_{\mathrm{z}} = E_{\mathrm{z}} - E_{\mathrm{F}} \ ; \tag{19}$$

then the current density is written as

$$j(\varepsilon) \sim \frac{e^{-G_{\mathrm{F}}}}{1 + e^{\varepsilon/pd_{\mathrm{F}}}} \int_{-\infty}^{\varepsilon} d\varepsilon_{\mathrm{z}} e^{\varepsilon_{\mathrm{z}}/d_{\mathrm{F}}} \ ; \tag{20}$$

where $p = k_B T/d_F$ is the normalized temperature ($p$ is lower than ~1 to approximately neglect the thermionic emission). By integrating Supplementary Equation (20) with $\varepsilon$ from -∞ to ∞, the influence of the temperature on the field emission is written as

$$J(T) = J_0 \frac{\pi p}{\sin \pi p} \ . \tag{21}$$

The constant $J_0$ is given by Supplementary Equation (17).

By using Supplementary Equation (21), the average total energy $\langle\varepsilon\rangle$ is calculated as [Supplementary Reference 6],

$$\langle \varepsilon \rangle = \frac{\int_{-\infty}^{\infty} \varepsilon j(\varepsilon) d\varepsilon}{\int_{-\infty}^{\infty} j(\varepsilon) d\varepsilon} = -d_{\mathrm{F}} \varphi(p) \ ; \tag{22}$$

where $\phi\,(p)$ can be approximately written as,

$$\varphi(p) \cong \pi p \cot \pi p \tag{23}$$

Similarly to Supplementary Equation (22), we calculate the average normal energy $\langle\varepsilon_z\rangle$ as (see Supplementary Equation (20))

$$\langle \varepsilon_z \rangle = \frac{\int_{-\infty}^{\infty} \frac{e^{-G_F} d\varepsilon}{1+e^{\varepsilon/(pd_F)}} \int_{-\infty}^{\varepsilon} \varepsilon_z e^{\varepsilon_z/d_F} d\varepsilon_z}{\int_{-\infty}^{\infty} j(\varepsilon) d\varepsilon} = -d_F\left[1+\varphi(p)\right] = -d_F + \langle \varepsilon \rangle \ . \quad (24)$$

Finally, the average transverse energy $E_T$ is obtained as the difference of $\langle \varepsilon \rangle$ and $\langle \varepsilon_z \rangle$,

$$E_T = \langle \varepsilon \rangle - \langle \varepsilon_z \rangle = d_F \ . \quad (25)$$

By noting

$$E_T = \left\langle \frac{1}{2m}\left(p_x^2 + p_y^2\right) \right\rangle \ ; \quad (26)$$

$$\sigma_{p,x} = \frac{\sqrt{\langle p_x^2 \rangle}}{m} = \frac{\sqrt{mE_T}}{m} = \sqrt{\frac{d_F}{mc^2}} \ ; \quad (27)$$

the intrinsic rms emittance of the field emission beam per unit source size is given by Supplementary Equation (27). In the case of $F_{tip}$ of 2-5 GV m$^{-1}$ and $\phi$ = 4.5 eV, $d_F$ is equal to 0.1-0.2 eV the intrinsic emittance of field emission beam is 0.45-0.63 μm/mm-rms.

In the case of the field emission of electrons excited by near infrared single photon with the photon energy of $\hbar\omega$, we can estimate its intrinsic emittance by replacing the work function by $\phi - \hbar\omega$: in the case of $\hbar\omega$ equal to 1.6 eV with the same $F_{tip}$ and $\phi$ as above, $d_F$ is equal to 0.12-0.25 eV, therefore, the emittance is ~10 % larger.

The increase of $F_{tip}$ leads to the increase of both the emission current and the emittance. However, owing to the exponential sensitivity of the field emission current on $F_{tip}$, the orders of magnitude increase of the emission current results in only a marginal increase of the emittance: 50 % increase of $F_{tip}$ from 4 GV m$^{-1}$ to 6 GV m$^{-1}$ results in the increase of $J$ by a factor of $4\times10^2$ and the increase of the emittance by 20 %.

Another interesting observation is the temperature dependence. Both averages of the total energy and the normal energy monotonically increase with the increase of the temperature but the temperature dependence of $E_T$ and the emittance is absent at least for $T$ below approximately 1500 K, which is valid for the field emission without contribution from thermionic emission.

## Supplementary Reference


1. Müller, M., Paarmann, A., & Ernstorfer, R., Femtosecond electrons probing currents and atomic structure in nanomaterials, Nature Communications **5**, 5292 (2014);

2. NIST Standard Reference Database 64, NIST Electron Elastic-Scattering Cross-Section, http://www.nist.gov/srd/nist64.cfm .

3. Young, R. D., Theoretical Total-Energy Distribution of Field-Emitted Electrons, *Phys. Rev*. **113**, 110-114 (1959);

4. Gadzuk, J. W., & Plummer, E. W., Field Emission Energy Distribution (FEED), *Rev. Mod. Phys*. **45**, 487-548 (1973);

5. Forbes, R. G, & Deane, J. H. B., Reformulation of the standard theory of Fowler–Nordheim tunnelling and cold field electron emission, *Proc. R. Soc*. A **463**, 2907–2927 (2007);

6. Swanson, L. W., Crouser, L. C., & Charbonnier, F. M., Energy Exchanges Attending Field Electron Emission, *Phys. Rev*. **151**, 327-340 (1966);
